# The role of cell adhesion molecules in visual circuit formation: From neurite outgrowth to maps and synaptic specificity

**DOI:** 10.1002/dneu.22267

**Published:** 2015-02-19

**Authors:** Mégane Missaire, Robert Hindges

**Affiliations:** ^1^ MRC Centre for Developmental Neurobiology, King's College London, New Hunt's House, Guy's Campus, London SE1 1ULUnited Kingdom; ^2^ Present address: Master BioSciencesDépartement de BiologieÉcole Normale Supérieure de LyonUniversité de LyonUCB Lyon1, 46 Allée d'ItalieLyonFrance

**Keywords:** cell adhesion molecules, visual system, topographic map, axon pathfinding, synaptic targeting

## Abstract

The formation of visual circuitry is a multistep process that involves cell–cell interactions based on a range of molecular mechanisms. The correct implementation of individual events, including axon outgrowth and guidance, the formation of the topographic map, or the synaptic targeting of specific cellular subtypes, are prerequisites for a fully functional visual system that is able to appropriately process the information captured by the eyes. Cell adhesion molecules (CAMs) with their adhesive properties and their high functional diversity have been identified as key actors in several of these fundamental processes. Because of their growth‐promoting properties, CAMs play an important role in neuritogenesis. Furthermore, they are necessary to control additional neurite development, regulating dendritic spacing and axon pathfinding. Finally, trans‐synaptic interactions of CAMs ensure cell type‐specific connectivity as a basis for the establishment of circuits processing distinct visual features. Recent discoveries implicating CAMs in novel mechanisms have led to a better general understanding of neural circuit formation, but also revealed an increasing complexity of their function. This review aims at describing the different levels of action for CAMs to shape neural connectivity, with a special focus on the visual system. © 2015 Wiley Periodicals, Inc. Develop Neurobiol 75: 569–583, 2015

## INTRODUCTION

The establishment of correct circuitry in the nervous system is a highly complex process involving many different steps. This includes the appropriate generation and positioning of individual cell types, neurite extension and axon pathfinding, target innervation, up to mechanisms that control the cellular and subcellular specificity of synaptic connections. For a long time, sensory systems, and in particular the visual system, have served as models to study the molecular mechanisms underlying the generation of fully functioning networks. Retinal ganglion cells (RGCs), the sole output neurons of the retina, project from the eye to their primary targets in the brain proper, where they form topographic connections. Superimposed onto this general arrangement of axonal projections by all RGCs are functionally discrete circuits (conveying information including motion, brightness or color), generated by subsets of neurons that can be distinguished by their specificity in synaptic connectivity, laminar targeting, and cellular distribution. While classical axon guidance molecules, such as Eph receptors and their ligands, the ephrins, have been shown to control large parts of axon pathfinding decisions and retinotopic map formation, proteins belonging to a different class, the cell adhesion molecules (CAMs), have been identified as major players in the other processes of circuitry formation.

**Figure 1:**
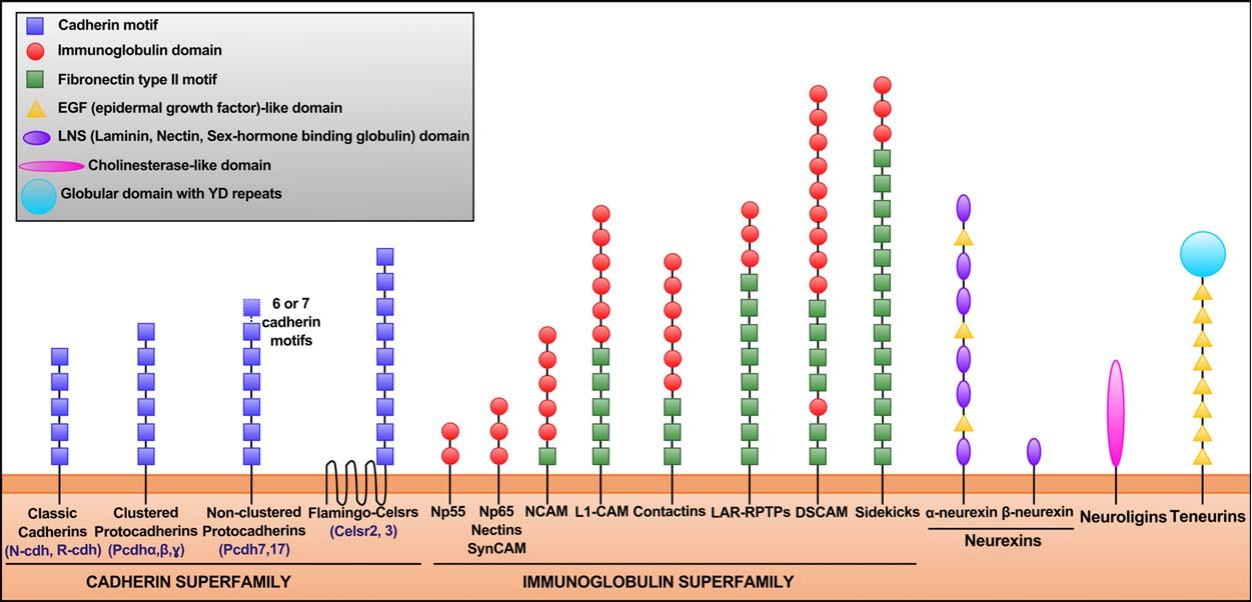
Structural diversity of CAMs. Schematic of the structural domains of CAMs. Two superfamilies of CAMs are involved in cell–cell adhesion: cadherins and immunoglobulins. Other families such as neurexins, neuroligins, and teneurins are also essential for cell–cell adhesion. The majority of these CAMs establish trans‐homophilic interactions, but they can also interact heterophilically in trans (e.g., neurexins‐neuroligins) or in cis (several CAMs such as N‐cadherin, NCAM, L1, or neuroplastins (Np) interact with FGFR). Some CAMs, such as NCAM or teneurins are found in dimers, and they can form cis‐clusters in the plasma membrane.

CAMs form a diverse group of transmembrane molecules implicated in cell–cell or cell‐extracellular matrix (ECM) interactions based on their homophilic and/or heterophilic adhesion properties. The main four CAM families studied to date are: cadherins, immunoglobulin superfamily cell adhesion proteins, integrins, and neurexins/neuroligins (Shapiro et al., 2007). However, several other families, including the recently discovered teneurins (Young and Leamey, 2009), are also regarded as adhesion molecules (Fig. 1). The CAMs involved in cell–cell adhesion are characterized by a high structural diversity, which reflects their vast functional diversity. Indeed, many CAMs display functions such as cell signaling (Shima et al., 2007; Hansen et al., 2008), cytoskeleton remodeling (Maness and Schachner, 2007; Hansen et al., 2008), or control of gene expression (Piper et al., 2008; Young and Leamey, 2009; Kleene et al., 2010). Genome‐wide association studies revealed that many CAMs are genetically linked to human psychiatric disorders, such as autism spectrum disorders, schizophrenia, bipolar disorder, mental retardation or depression (Maness and Schachner, 2007; Hirano and Takeichi, 2012; Hong et al., 2012; Krueger et al., 2012). Conversely, recent structural and functional imaging studies have shown aberrant neural connectivity patterns throughout the brains of patients with mental illnesses (Meyer‐Lindenberg, 2010; Fornito et al., 2012; Tost et al., 2012). Together, although there are many genes that could be causative for functional and structural disconnection of circuits, CAMs are prominent candidates where mutations could lead to different psychiatric disorders. They are therefore subject to intense research in a variety of systems and species.

In this review, we will give an overview of the different roles of CAMs and their function during different steps of visual system development: from the initial generation of neurites after RGC differentiation to RGC axon extension toward their targets, thereby passing several choice points along their way, and finally to the subsequent mapping within these areas according to topographic principles. During maturation of visual circuit formation, RGCs form synapses with specific presynaptic and postsynaptic partners in the retina and the tectum/superior colliculus (SC), respectively. A general principle in organizing connections between functionally similar classes of neurons is their arrangement in laminae. And, although we are still far from a complete understanding of the molecular determinants of synaptic laminar specificity, CAMs have been shown to play essential roles in this process in multiple species (Huberman et al., 2010; Sanes and Zipursky, 2010; Baier, 2013).

The role of one specific CAM in the whole formation of a neural network (Hirano and Takeichi, 2012) or the cooperative roles of different CAMs for one precise step of this process (Krueger et al., 2012) have been reviewed previously. In contrast, this review aims to provide a wider view of the functional diversity that CAMs have during the different steps of visual system development, including the formation of the topographic map.

## CAMs AND NEURITE OUTGROWTH

**Figure 2:**
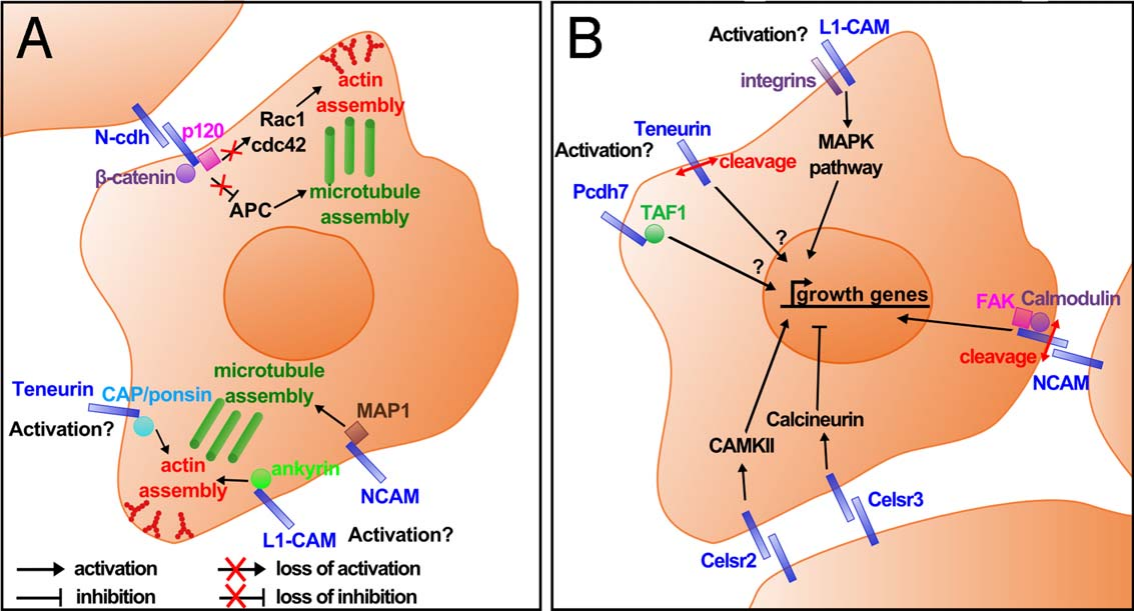
CAMs can activate neurite outgrowth through different mechanisms. The two subsets represent a neuroblast, surrounded by other neurons, undergoing neurite outgrowth. (A) Mechanical activation of neurite outgrowth by CAMs through cytoskeleton remodeling, which is necessary for membrane protrusion. N‐cadherin (N‐cdh) is activated by trans‐homophilic interaction, and can therefore bind β‐catenin and p120 catenin. N‐cadherin is bifunctional because of its growth‐promoting action through β‐catenin and activation of microtubule assembly, and growth‐inhibiting action through p120 and inhibition of actin assembly. The mode of activation of teneurins, L1, and NCAM is still unknown, but they all foster cytoskeleton remodeling through their intracellular partners. (B) Genetic activation of neurite outgrowth by CAMs. L1, Celsr2, and Celsr3 activate the MAPK pathway, CAMKII, and calcineurin, respectively, to modulate gene expression. Celsr2 and Celsr3 are activated by trans‐homophilic interaction, but how integrin‐binding to L1 is induced is still unclear. The cytosolic cofactor TAF1 of Pcdh7 (NF‐protocadherin) might translocate into the nucleus to activate growth in response to an unknown signal. The ICDs of teneurins and NCAM are cleaved and might modulate transcriptional activity in the nucleus. The signal of cleavage is only known for NCAM and corresponds to its trans‐binding.

After their differentiation, neurons migrate to their appropriate location, where they undergo neuritogenesis and begin to generate axon and dendrites, characteristic of mature neurons. The interaction with the ECM is crucial during the process of axon outgrowth. For example, in the visual system it has been shown that functional inhibition of the CAM integrin leads to general impairment of neurite outgrowth in RGCs *in vivo* (Lilienbaum et al., 1995). We will focus here on two types of mechanisms by which CAMs can promote neurite outgrowth: cytoskeleton remodeling and modulation of gene activation (Fig. 2), including their affected cell signaling pathways.

### CAMs and Cytoskeleton Remodeling During Neurite Outgrowth

Neuritogenesis requires the reorganization of the neuronal cytoskeleton and CAMs are important for triggering this process, for example, through their interaction with catenins. Cytoskeleton remodeling establishes the structure of the growth cone, which is composed of actin filaments necessary for membrane protrusion, and microtubules in the central area required for neurite extension (Geraldo and Gordon‐Weeks, 2009). Neurite extension itself is positively regulated by the adenomatous polyposis coli (APC) protein, which in turn can be inhibited by β‐catenin (Votin et al., 2005). N‐cadherin was shown to promote neurite outgrowth through sequestration of β‐catenin, therefore releasing the inhibition of APC action (Hansen et al., 2008) [Fig. 2(A)]. However, it also has been shown that N‐cadherin can have a growth inhibiting effect through binding to the cytoplasmic p120 catenin, which then is prevented from activating actin remodeling through the GTPases Cdc42 and Rac1 (Noren et al., 2000). In this case, N‐cadherin can prevent excessive neurite outgrowth at focal locations [Fig. 2(A)]. The interaction between cadherins and catenins is regulated by phosphorylation through different kinases, such as Fyn and Src (Lilien and Balsamo, 2005). However, the exact regulation between growth promoting and inhibiting functions is not clear (Hansen et al., 2008). In the *Xenopus* visual system, the expression of a dominant‐negative form of N‐cadherin in RGCs leads to a significant reduction in both initial neurite outgrowth and subsequent axonal elongation along the entire optic pathway, thereby confirming N‐cadherin as a growth‐promoting molecule *in vivo* (Riehl et al., 1996).

Two members of the immunoglobulin superfamily have also been shown to promote cytoskeleton remodeling. NCAM binds tubulin and microtubule‐associated protein‐1 (MAP1) to foster microtubule polymerization (Buttner et al., 2003), whereas L1 can remodel the actin cytoskeleton via Spectrin (Maness and Schachner, 2007).

Another CAM family that has been shown to interact or remodel the cytoskeleton consists of the teneurins. The intracellular domain (ICD) of Teneurin‐1 was shown to interact with the cytoskeleton adaptor protein CAP/ponsin, which itself binds to multiple factors, such as Cbl or focal adhesion kinase (FAK), regulating cell–cell adhesion and the actin cytoskeleton (Ribon et al., 1998; Scaife and Langdon, 2000; Nunes et al., 2005) [Fig. 2(A)]. Moreover, both Teneurin‐1 and −2 are anchored via their ICD to the actomyosin cytoskeleton, which is necessary for strengthening of cell–cell adhesions and thus results in a reduction of neurite outgrowth (Beckmann et al., 2013). Furthermore, recent studies carried out in *Drosophila* have demonstrated that teneurin perturbations lead to a disorganization of microtubules in presynaptic terminals, as well as a disruption of the Spectrin cytoskeleton on the postsynaptic side (Mosca et al., 2012).

In summary, different families of CAMs have been shown to directly or indirectly influence the organization of the cytoskeleton, which in turn has profound effects on neurite outgrowth, branch formation, or even synaptogenesis.

### Neurite Outgrowth Through Gene Activation by CAMs

Transcriptional regulation is essential during neurite outgrowth for the synthesis of new membrane components and proteins. A genome‐wide RNAi screen in *Drosophila* identified a large number of genes important for neurite outgrowth, including transcription factors, cytoskeleton proteins and CAMs (Sepp et al., 2008).

CAMs play a key role in genetic activation of neurite outgrowth through several pathways. For instance, L1 activates the MAPK pathway by recruiting integrins, and therefore, modifies gene expression (Maness and Schachner, 2007) [Fig. 2(B)]. However, it is unclear whether this recruitment is triggered by *cis* or *trans* interactions of L1. The mammalian seven‐pass transmembrane cadherins Celsr2 and Celsr3, orthologues of the *Drosophila* Flamingo protein, modulate neurite outgrowth through the activation of CAMKII (calcium/calmodulin‐dependent protein kinase II) or calcineurin (Shima et al., 2007). Interestingly, Celsr2 and Celsr3 activation have opposing effects on neurite outgrowth. To mimic trans‐homophilic binding, purified recombinant cadherin‐domain repeats of Celsr2 and Celsr3 were applied to dissociated hippocampal primary neurons. These experiments showed that Celsr2 fosters a large calcium influx and thereby activates CAMKII leading to a growth‐promoting effect. In contrast, the calcium influx triggered by Celsr3 is smaller, thus activating calcineurin, which then leads to neurite growth inhibition (Shima et al., 2007) [Fig. 2(B)]. As a result, this system using two possibly cooperating cadherins would be able to finely balance appropriate neurite outgrowth.

Another mechanism through which gene expression is regulated is the translocation of CAM cytosolic partners into the nucleus. In the *Xenopus* retina, NF‐protocadherin (Pcdh7) and its cytosolic cofactor template‐activating factor 1 (TAF1) were shown to be necessary for neurite outgrowth of RGCs, and TAF1 was suggested to regulate gene expression in the nucleus (Piper et al., 2008) [Fig. 2(B)]. Furthermore, a previously unreported growth‐promoting action of NCAM was shown in *in vitro* experiments, through the translocation of a fragment of the adhesion molecule itself into the nucleus (Kleene et al., 2010). Indeed, after its trans*‐*homophilic binding, NCAM is recruited and dimerized in lipid rafts, where calmodulin and FAK subsequently bind to the NCAM ICD. After the cleavage of the extracellular domain of NCAM, its ICD and FAK translocate in a calmodulin‐dependent way into the nucleus, where they possibly interact with transcription factors, triggering the expression of neurite outgrowth‐promoting genes (Kleene et al., 2010) [Fig. 2(B)]. In a similar fashion, the ICDs of Teneurin‐1 and Teneurin‐2 have been shown to translocate into the nucleus after proteolytic release from the membrane (Bagutti et al., 2003; Nunes et al., 2005; Kenzelmann et al., 2008). It is suggested that this transport is mediated through a putative nuclear localization signal in the ICDs of teneurins (Kenzelmann et al., 2008). In the nucleus, the ICD of Teneurin‐1 interacts with the transcriptional repressor MBD1, a member of the methyl‐CpG‐binding domain family of proteins, in addition to the aforementioned adapter protein CAP/ponsin (Nunes et al., 2005). However, the exact signal triggering the proteolytic cleavage of teneurins, including the identity of proteases involved, are still unknown.

Taken together, CAMs play an essential role in the regulation of neuritogenesis through different but complementary pathways. These pathways include direct interaction with cytoskeletal proteins at the membrane as well as indirect action through nuclear activation of transcription factors. An overview, listing some of the downstream molecules of CAMs, is given in Table 1.

**Table 1:** List of Molecules Acting Downstream Cell Adhesion Molecules

Type/Family	Name	General Cellular Role	Upstream CAM	References
Cytoskeleton proteins	G‐actin	Free monomers of G‐actin form microfilaments of F‐actin, which are part of the cytoskeleton and involved in multiple cellular processes (cell division, vesicle traffic…)	N‐cadherin, L1, Teneurins	Maness and Schachner (2007); Hansen et al. (2008); Beckmann et al. (2013)
	Tubulin	Dimeres of α‐ and β‐tubulin assemble to form microtubule filaments, which are part of the cytoskeleton and involved in multiple cellular processes (cell division, vesicle traffic…)	NCAM	Buttner et al. (2003)
	Spectrin	Forms a network on the intracellular side of the plasma membrane to maintain its integrity, and links the actin filaments together to maintain the cytoskeleton structure	L1	Davis and Bennett (1994)
Cytoskeleton associated proteins	MAP1 (microtubule associated protein)	Enhances microtubule assembly by stabilizing microtubules filaments	NCAM	Buttner et al. (2003)
	CAP (Cbl associated protein)/ponsin	Adaptor protein involved in the regulation of the actin cytoskeleton and cell adhesion	Teneurin‐1	Nunes et al. (2005)
Catenins	β‐catenin	Links N‐cdh to F‐actin via α‐catenin	N‐cadherin	Riehl et al. (1996)
	p120‐catenin	Stabilizes N‐cdh to the plasma membrane	N‐cadherin	Reynolds et al. (1996)
Rho GTPases, signaling G proteins	Cdc42	Regulates signaling pathways involved in cell cycle, cell morphology, cell migration, or endocytosis	N‐cadherin	Noren et al. (2000)
	Rac1	Regulates signaling pathways involved in cell cycle, cell–cell adhesion, or motility	N‐cadherin	Noren et al. (2000)
Kinases	MAPK (mitogen‐activated protein kinase)	The MAPK phosphorylation cascade is involved in multiple signaling pathways to regulate cellular functions such as gene expression, cell division, apoptosis, or differentiation	L1	Maness and Schachner (2007)
	CAMKII (Ca2+/calmodulin‐dependent protein kinase II)	Is involved in multiple signaling pathways especially involved in learning and memory, and can modulate gene expression via transcription factor regulation	Celsr2	Shima et al. (2007)
	FAK (Focal adhesion kinase)	Is concentrated in focal adhesion to enhance cell migration by regulating cell adhesion	NCAM	Kleene et al. (2010)
Phosphatase	Calcineurin	Modulates gene expression by activating the transcription factor NFAT	Celsr3	Shima et al. (2007)
Calcium‐binding messenger protein	Calmodulin	Intermediate messenger transducing calcium signals and involved in many cellular processes such as apoptosis, metabolism or movements of organelles	NCAM	Kleene et al. (2010)
Transcriptional repressor	MBD1 (Methyl‐CpG‐binding domain protein 1)	Binds to methylated sequences of DNA, and can in particular repress the transcription of genes with a methylated promoter	Teneurin‐1	Nunes et al. (2005)

## CAMs AND NEURITE DEVELOPMENT

The axon and dendrites formed during neurite outgrowth extend and project to their appropriate targets where they then form specific connections with their synaptic partners. On their way, axons encounter several major choice points where the growth cone has to make guidance decisions for the correct continuation of growth.

### CAMs During Axon Pathfinding and Target Selection

Axons formed during neuritogenesis extend toward their target in the CNS in multiple steps. However, this growth is not random and ensures the functionality of the CNS through the formation of appropriate connections between neurons. CAMs act in addition to classical axon guidance molecules at different steps of circuitry formation most likely through specific contact adhesion.

It has been shown that CAMs can have directional growth‐promoting action for neurites. For instance, the trans‐homophilic interaction of R‐cadherins located on of mouse forebrain pioneer axons and on the substrate promotes axon outgrowth, favoring therefore an extension of the pioneer axons toward high concentrations of R‐cadherin (Andrews and Mastick, 2003). A similar effect was observed for N‐cadherin (cdh2) in zebrafish, where the protein is required to elicit stereotypic turns that guide axons of cranial sensory ganglia neurons from their intermediate to their final targets (LaMora and Voigt, 2009).

In the retina, it was shown that several CAMs are essential for the correct extension of axons toward the exit point of the eye. Blocking the functions of L1, NrCAM or neurolin (also called BEN/DM‐GRASP/ALCAM) leads to RGC axon fasciculation defects and subsequent errors in directed growth toward the optic disk (Brittis and Silver, 1995; Ott et al., 1998; Weiner et al., 2004). The next step of the RGC axons journey is the exit from the eye through the optic disk. This mechanism has been shown to depend on at least two opposing forces. On one side, RGC axons are pushed away from the retinal periphery through inhibitory signaling mediated by a central‐peripheral gradient of chondroitin sulfate proteoglycans (Brittis et al., 1992). On the other hand, RGC axons express the receptor deleted in colorectal cancer (DCC), which mediates strong attraction to Netrin‐1 released by optic disk glia (Deiner et al., 1997). In Netrin‐1 and DCC mutants, although RGC axons are generated and extend away from the periphery, they fail to exit the retina at the disk, leading to an optic nerve hypoplasia (Deiner et al., 1997).

Once RGC axons have exited the retina, they form the optic nerve, which extends toward the next major guidance choice point, the optic chiasm. Dependent on the lack or presence of binocular vision (i.e., animals with various degrees of visual overlap between the two eyes), the axonal projection will either fully cross the mildine or exhibit partial crossing with contralateral and ipsilateral trajectories, respectively (Erskine and Herrera, 2007). The deflection of ipsilaterally projecting axons at the chiasm is mediated by a repulsive interaction of the receptor tyrosine kinase EphB1, expressed in RGCs, and its ligand ephrin‐B2, expressed by the midline glia (Williams et al., 2003). In the retina, EphB1 expression is controlled by the transcription factor Zic2 whose expression domain is tightly linked to the area of visual overlap between the eyes (Herrera et al., 2003; Garcia‐Frigola et al., 2008). In mouse, this region is called the ventral‐temporal crescent, where Zic2 expression is regulated by the LIM‐homeodomain transcription factor Isl2 (Pak et al., 2004). Recently, it has been shown that mutations in Teneurin‐2 (Ten‐m2) lead to a downregulation of EphB1 in mouse and a subsequent decrease of ipsilaterally projecting RGC axons, while Zic2 expression remains unaltered (Young et al., 2013). Interestingly, earlier reports showed that Ten‐m2 attenuates the transcriptional activity of a different member of the Zic family, Zic1, *in vitro* (Bagutti et al., 2003). It is therefore plausible that Ten‐m2 similarly decreases the transcriptional activity of Zic2, thus leading to a reduced expression of EphB1. Indeed, an impairment of Zic2 transcriptional activity has been found for Teneurin‐3 (Ten‐m3) *in vitro* (Chun and Hindges, unpublished results). Ten‐m3 is required for appropriate mapping of ipsilateral, but not contralateral projections from the retina to the dLGN and is therefore necessary for the generation of binocular maps in mice (Leamey et al., 2007; Dharmaratne et al., 2012). The exact molecular mechanisms for these functions are still unclear, as none of the teneurins exhibit a clear expression pattern that is specific for either the ipsilateral or contalateral RGC population in the retina (Young and Leamey, 2009). It is, however, conceivable that teneurins interact with specific molecular components that regulate laterality and mapping of projections. Molecular interaction studies for different teneurins should shed some light on this in the future.

In addition to repellent actions for ipsilaterally projecting RGC axons, positive cues exist that are critical for RGCs axons to cross the midline. NrCAM is expressed by the contralateral projecting RGC population, as well as the midline glia at the chiasm, and a mutation in the gene leads to pathfinding defects at the mouse optic chiasm (Williams et al., 2006). Recently, it was further shown that NrCAM does not act alone, but rather in combination with Sema6D and Plexin‐A1 to enable contralateral projections and thereby to control correct decussation at the optic chiasm (Kuwajima et al., 2012).

**Figure 3:**
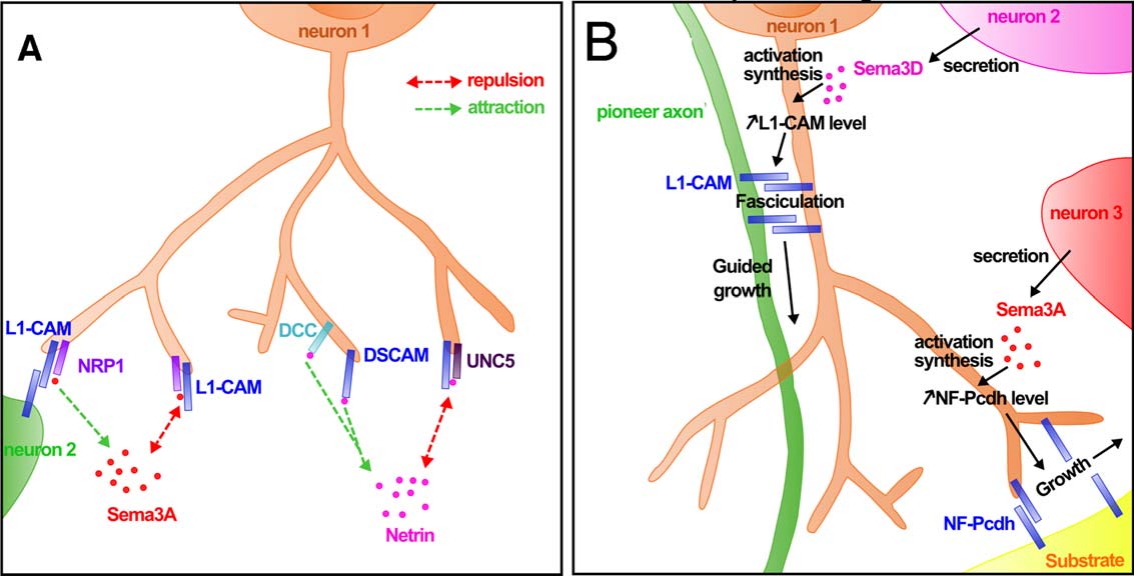
CAMs and axon pathfinding mechanism. (A) CAMs can be receptors for guidance cues. DSCAM can cooperate with UNC5 to induce repulsion in response to netrin, or with DCC to induce attraction toward this guidance cue. DSCAM and UNC5 physically interact for Netrin binding, contrary to DSCAM and DCC. Similarly, L1‐CAM and NRP1 (and PlexinA4 and TAG1 which are not represented) form a bifunctional complex receptor for Sema3A. Indeed, this complex induces repulsion in response to Sema3A, but when L1‐CAM makes trans‐homophilic interaction, this repulsion is turned into attraction. (B) CAMs can act as guidance cues. In response to the secretion of Sema3D by neuron 2, the membrane level of L1‐CAM is increased in the axon of neuron 1. As a result, fasciculation is promoted by the trans‐homophilic interaction of L1‐CAM between neuron 1 and a pioneer axon. In the same way, Sema3A secreted by neuron 3 can activate the synthesis of NF‐Protocadherin (NF‐Pcdh) in neuron 1, triggering its trans‐homophilic adhesion, and the oriented growth on a substrate.

Interestingly, CAMs can act also as coreceptors for guidance cues [Fig. 3(A)]. For example, using cocultures of mouse spinal neurons, it was demonstrated that L1 is able to form a complex with neuropilin1 to mediate the repulsive action by Sema3A (Castellani et al., 2000). Interestingly, the authors further showed that soluble L1 can also convert the repulsive action of Sema3A into attraction by interacting in trans with neuropilin‐1, therefore acting as a mediator balancing these two opposing activities.

Moreover, expression of CAMs themselves can be regulated by other guidance cues to orient axon growth [Fig. 3(B)]. For instance, using the medial longitudinal fascicle in zebrafish as a model, it was shown that Sema3D, which is usually considered as a repulsive molecule, can promote axon fasciculation through CAM‐mediated processes. Fasciculation allows the axons to follow an already established tract toward their target and is essential for axon pathfinding. Sema3D indeed increases the membrane localization levels of L1 and, therefore, activates cell–cell adhesion with pioneer axons (Wolman et al., 2007). The mechanism by which Sema3D regulates the L1 protein level, however, is still unclear. Similarly, in the *Xenopus* optic tract, Sema3A activates the synthesis of NF‐protocadherin to promote RGC axon growth toward the optic tectum (Leung et al., 2013). In both cases, the increased expression of the CAMs reinforces cell–cell adhesion, which is necessary for the axon to adhere to its substrate. Interestingly, overexpression of Ten‐m3 in dissociated cultures induces neurite fasciculation, and mice that contain a mutation in this gene exhibit defasciculation of RGC axons along the visual pathway (Symonds and Hindges, unpublished results), therefore suggesting a role of this protein in axon–axon interactions.

A recent report describes the involvement of cadherins in visual target selection (Osterhout et al., 2011). The authors show that in mice, cadherin‐6 (Cdh6) is expressed by a subset of RGCs and their targets in the brain, which are all part of the nonimage forming nuclei. Deletion of Cdh6 leads to a failure of these RGCs to innervate their appropriate nuclei and instead leads to a mis‐projection to other visual targets (Osterhout et al., 2011). Although the precise mechanisms are not clear yet, it is more likely that these defects are based on homophilic (or heterophilic) interactions between Cdh6‐expressing RGCs and their postsynaptic partners rather than through mechanisms affecting axon–axon interactions, as mutant mice did not exhibit any defasciculation defects in their misrouted RGCs axon projections.

### CAMs in Topographic Map Formation

The formation of the retinotopic map, where the neighbor relationship of RGCs in the retina is preserved in the arrangement of their projections within their main midbrain target—the optic tectum of fish, amphibian, and birds, or the SC in mammals—is realized through a combination of molecular cues and activity‐dependent mechanisms (Feldheim and O'Leary, 2010). As initially postulated by Sperry, the molecular control is based on the graded expression of interacting chemical cues in the origin and target areas (Sperry, 1963). Although the mapping of the nasal‐temporal retinal axis is determined by opposing gradients of EphA receptors and ephrin‐A ligands in the retina and SC mediating repulsion, the correct projections of RGC axons originating along the dorsal‐ventral retinal axis is dependent mainly on the graded expression of EphBs and ephrin‐Bs acting as bifunctional molecules to mediate attraction and repulsion, in combination with a repulsive activity by Wnt‐Ryk signalling (Hindges et al., 2002; McLaughlin et al., 2003; Schmitt et al., 2006; Feldheim and O'Leary, 2010). CAMs have been shown to act as additional factors controlling topographic map formation. Mice lacking the adhesion molecule L1 were shown to develop mapping defects along both axes of the SC (Demyanenko and Maness, 2003). However, L1 is localized on RGC axons only, without apparent gradients along the two axes, suggesting a mechanism in mapping that is not based on homophilic interactions. Interestingly, while null mutants of L1 exhibit more pronounced defects along the anterior‐posterior SC axis compared to the medial‐lateral axis, a point mutation in L1 abolishing binding to the cytoskeleton adaptor protein ankyrin leads to strong defects along the latter, suggesting a functional linkage to the EphB/ephrin‐B system (Buhusi et al., 2008). Indeed, recent data show that EphB receptors are able to phosphorylate L1 and the closely related family member NrCAM at their ankyrin‐binding motifs, thereby modulating this interaction important for medial‐lateral topographic mapping (Dai et al., 2012, 2013). In addition, the activated leukocyte CAM ALCAM (BEN/SC‐1/DM‐GRASP/Neurolin) is expressed in the SC during RGC axon ingrowth and ALCAM null mutant mice also exhibit defects in mediolateral map formation (Buhusi et al., 2009). *In vitro* experiments in the same study further suggest that this effect is based on the trans‐heterophilic interaction between L1 on RGC axons and ALCAM on collicular cells, thereby promoting cell adhesion for medial branch extension.

Finally, as mentioned earlier, it is important to note that Sema3D can influence the expression of L1 and lead to an increase in adhesion (and thus fasciculation) between axons (Wolman et al., 2007). The involvement of semaphorins and their receptors plexins/neuropilins in RGC outgrowth and mapping (Campbell et al., 2001; Liu et al., 2004; Claudepierre et al., 2008) therefore suggest the possibility of a functional crosstalk between these molecules and CAMs, critical for the correct formation of the overall retinotopic map.

## CAMs IN SYNAPTIC SPECIFICITY AND FUNCTIONAL VISUAL CIRCUIT FORMATION

In addition to the roles during the formation, outgrowth, and main target selection of neurites, CAMs play essential roles in the finer details of circuit formation, including lamina‐specific targeting, formation of synapses, cell type‐specificity of synapses, and finally self‐avoidance mechanisms for neurons. As these are vast and intensely researched fields, we will summarize here the most important points in the context of visual system only and point out additional review articles for these different subjects, where possible.

### CAMs and Dendrite Self‐Avoidance

After neurite outgrowth, the extension of dendrites creates dendritic arborizations, which can be organized in isoneural (self‐avoidance) and heteroneural (tiling) spacing. These mechanisms allow the arbors to maximize their coverage and to avoid redundant inputs caused by branch overlaps. Self‐avoidance is characterized by the repulsion between dendrites of a single neuron, whereas tiling consists in the repulsion between dendrites of two different but functionally related neurons. These avoidance processes require selective recognition and repulsion, and a molecular code that defines “self” versus “nonself” (Grueber and Sagasti, 2010). In *Drosophila*, it was shown that the Ig‐SF CAM Down syndrome cell‐adhesion molecule 1 (Dscam1) can act as a regulator of self‐avoidance (Schmucker et al., 2000). This large protein undergoes extensive alternative splicing that can generate up to 19,008 different extracellular isoforms connected to one of two alternatively spliced transmembrane domains, therefore, bringing the total number of possible isoforms to 38,016. Individual neurons stochastically express a unique combination of isoforms, therefore, differentiating them from other neurons (Miura et al., 2013). Binding assays showed that Dscam1 establishes almost exclusively isoform‐specific trans‐homophilic interactions (Wojtowicz et al., 2004). Therefore, on dendrites of the same neuron identical Dscam1 isoforms are presented that are able to interact and promote repulsion. Conversely, if the encountering dendrites come from unrelated neurons, the nonidentical Dscam1 isoforms do not bind to each other, thereby allowing neurite overlaps due to a lack of repulsion (Matthews et al., 2007; Grueber and Sagasti, 2010). The other member of the Dscam family in *Drosophila*, Dscam2, is also alternatively spliced, albeit to a lesser extend, and has been shown to mediate not only self‐avoidance but in addition also cell‐type specific avoidance (Millard et al., 2007; Lah et al., 2014).

In vertebrates, two Dscam genes are found, Dscam and Dscam‐like 1 (Dscaml1) and studies in the mouse retina have shown that the proteins act as a regulator of cell and neurite spacing, similar to the *Drosophila* Dscams (Fuerst et al., 2008; Fuerst et al., 2009). Mouse mutants for Dscam and Dscaml1 exhibit clumping of several cell types in the retina and fasciculation of their dendrites, including RGCs, suggesting a prominent function in self‐avoidance (Fuerst et al., 2009). However, vertebrate Dscams do not undergo extensive alternative splicing and it is suggested that their role is to generally mask existent adhesive cues between different types of retinal cells, rather than to promote specific repulsion through the generation of different isoforms.

Recent findings have shown in vertebrates that the family of protocadherins is responsible for the molecular emergence of dendritic self‐avoidance and the ability to discriminate between “self” and “nonself” (Lefebvre et al., 2012). In mouse, the protocadherin locus comprises 58 genes, arranged in three subclusters. Single neurons, including amacrine cells in the mouse retina, express different members of these subclusters in a probabilistic and combinatorial fashion, therefore generating high diversity between cells with different adhesion properties (Lefebvre et al., 2012; Thu et al., 2014). Mutant animals lacking an entire subcluster of protocadherins exhibit a loss of dendritic self‐avoidance in amacrine cells, as well as in cerebellar Purkinje cells. The authors further show that the introduction of a single protocadherin isoform into the subcluster mutant background is able to restore dendritic self‐avoidance of individual cells in the retina and the cerebellum (Lefebvre et al., 2012).

In summary, it becomes apparent that the significant mechanism of neuronal self‐avoidance is clearly conserved between invertebrates and vertebrates and is mediated by multiple families of CAMs. Interestingly, individuality between different cells as a prerequisite for the recognition of self versus nonself is achieved in both cases by the generation of different protein isoforms. However, they use different genes: while *Drosophila* is depending on Dscams, the mammalian system uses the structurally unrelated protocadherins and uses its Dscam proteins in cellular avoidance through different mechanisms.

### CAMs, Laminar Targeting, and the Specification of Functional Circuits

The coverage of visual space in form of topographic maps ensures the correct spatial representation of the world in the brain. Visual information, however, is preprocessed already in the retina and separated in parallel channels, encoding features such as motion or contrast. These functionally distinct circuits are established by different cell‐types, present in the retina and its target areas. Therefore, to set up this hierarchy of connectivity appropriately, cells have not only to follow the general rules of topographic mapping, but they also need to be able to generate cell type‐specific connections with their presynaptic and postsynaptic partners. A general organizational principle of synaptic connections between cells belonging to functionally identical (or at least similar) classes is the formation of individual laminae. The visual system is a prominent example exhibiting a layered organization in the nervous system (Sanes and Zipursky, 2010). It is estimated that the vertebrate retina consist of more than 100 different cell types, that can be morphologically and/or functionally distinguished (Baier, 2013). In the inner plexiform layer (IPL) of the retina, which lays between the RGC layer and the inner nuclear layer, bipolar cells, amacrine cells, and RGCs form specific synaptic connections, arranged in approximately ten individual laminae in mammals (Roska and Werblin, 2001), sometimes also combined as five major sublaminae S1–S5. It has been shown that CAMs play an essential role in the establishment of this cell type‐specific connectivity in the IPL.

**Figure 4:**
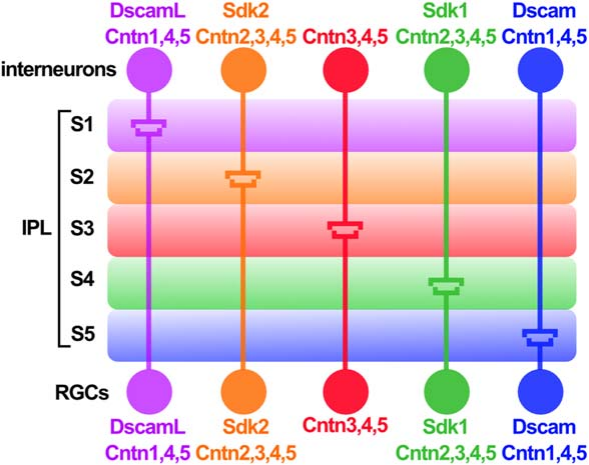
CAMs are essential for synaptic targeting. Example of the role of CAMs for synaptic targeting in the IPL of the chick retina. The amacrine and bipolar cells in the inner nuclear layer establish synapses with RGCs which express the same isoform combination of Dscams, Sidekicks (Sdk), and Contactins (Cntn). These synapses are formed in a specific layer of the IPL, S1–S5.

In the chick retina, it was found that four members of the Ig‐SF family, Dscam, DscamL, Sidekick‐1 and 2, are expressed by nonoverlapping groups of amacrine cells and RGCs. In each sublamina of the IPL, specific synapses are formed between neurons that match the expression of only one of these four Ig‐SF molecules. Misexpression of any of these proteins in cells that do not endogenously express that particular protein, drives their synaptic targeting into a different laminae in which the corresponding protein is found. Given that these CAMs establish strict homophilic adhesions *in vitro* and promote laminar specificity, they can act as matching cues to foster specific synaptic targeting (Yamagata et al., 2002; Yamagata and Sanes, 2008). However, the complexity and high number of interactions between functionally different cells in this system predicts the existence of additional molecules as part of this molecular code. Indeed, through gain‐ and loss‐of‐function analyses, contactins, a related family of Ig‐CAMs, were identified to be crucial for correct synaptic laminar targeting (Yamagata and Sanes, 2012) (Fig. 4). Interestingly, the aforementioned studies investigating Dscam or Dscaml1 in mice did not find alterations in the organization of retinal synaptic laminae, suggesting that the roles of Dscams in synaptic adhesion and specificity are not conserved in mammals (Fuerst and Burgess, 2009).

Several recent reports have combined the molecular identification of connectivity with the functionality of visual circuits. In zebrafish, Teneurin‐3 (*tenm3*) is necessary for synaptic targeting of RGCs subtypes not only in the IPL, but also in the main axonal target area, the optic tectum. The authors showed that a knockdown of *tenm3* leads to structural defects of RGC connectivity and further using functional optical imaging that this induces specific functional defects affecting orientation‐selectivity, without impairing direction‐selectivity (Antinucci et al., 2013). This suggests that *tenm3* provides the molecular information in specific cell types along the visual pathway to control the generation of a functionally distinct circuit. In mouse, it has been shown recently through gain‐ and loss‐of‐function approaches that two members of the type II cadherins, Cdh8 and Cdh9, are essential for the generation of direction selectivity in the retina by specifying the laminar connectivity of bipolar cells with RGCs (Duan et al., 2014). Deletion of either gene resulted in arborization defects of specific bipolar cells in the retinal IPL. Conversely, ectopic expression of Cdh8 or Cdh9 in amacrine cells that is usually negative for these genes lead to a displacement of their IPL arbors into areas typical for Cdh8‐ and Cdh9‐positive bipolar cells, respectively (Duan et al., 2014). All structural defects were accompanied by functional defects in direction‐selective visual responses. Interestingly, the study suggested that Cdh8 and Cdh9 act heterophilically, rather than through the typical homophilic interaction mechanism between cadherins. Although the exact binding partners for Cdh8 and Cdh9 in this system are still unknown, these findings are a good indication that the possible mechanisms of molecular interaction between different cells types are much wider than previously thought.

The role of CAMs in synaptic targeting is not restricted to the vertebrate visual system. Indeed, in the fly, N‐cadherin and the member LAR of the LAR‐RPTPs (Leukocyte common antigen‐related receptor protein tyrosine phosphatase) cooperate to regulate the layer‐specific targeting of the photoreceptor neurons in the optic lobe (Nern et al., 2008; Prakash et al., 2009). Furthermore, it was shown that teneurins instruct synaptic partner matching in the olfactory circuit as well as at the neuromuscular junction in *Drosophila* through trans‐synaptic homophilic adhesion (Hong et al., 2012; Mosca et al., 2012).

## CONCLUSIONS

**Figure 5:**
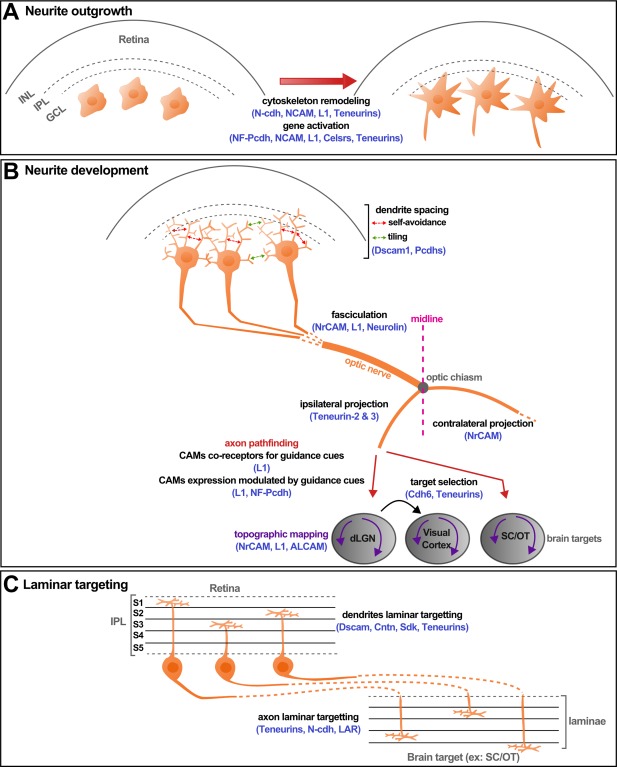
CAMs in visual system development. (A) Role of CAMs in RGCs neurite outgrowth. On this schematic representation of the retina, three RGCs undergo a CAM‐dependant neurite outgrowth via the two mechanisms detailed in the main text: cytoskeleton remodeling and gene activation. Examples of CAMs involved are written in blue. (B) Role of CAMs in RGCs neurite development. On one side of the RGCs, the dendritic arborization in the IPL is organized through self‐avoidance and tiling mechanisms. On the other side, RGC axons exit the retina and fasciculate to form the optic nerve that extends toward the optic chiasm, where (in case of binocular vision) some axons project ipsilaterally and others cross the midline to project contralaterally. After this choice point, RGC axons select their appropriate targets and project toward them, for example the SC/OT. This projection within these targets is organized by topographically to preserve the neighbor relationship of RGCs in the retina. Several CAMs (indicated in blue) are necessary for all these steps along the visual pathway. (C) Role of CAMs in RGC laminar targeting. The dendrites of the RGCs establish synapses in one or several specific sublaminae of the IPL with amacrine cells and bipolar cells (nonrepresented). In a similar fashion, the RGC axon can establish synapses in specific laminae in the brain target. Here again, CAMs (in blue) are involved in the specification of laminar targeting. dLGN, dorsal lateral geniculate nucleus; GCL, ganglion cell layer; INL, inner nuclear layer; IPL, internal plexiform layer; S1–S5, five sublaminae of the IPL; SC/OT, superior colliculus/optic tectum.

CAMs are key molecules in multiple steps of neural circuit formation. The visual systems of both invertebrates and vertebrates have been excellent models to elucidate the diverse functions of CAMs in neurite formation, axon pathfinding, and the development of topographic maps (Fig. 5). Recent findings place CAMs in the center for the regulation of synaptic targeting and specificity, resulting in distinct circuits for visual function and behavior. Although significant advances have been made to shed light onto the combinatorics of CAM expression and localization in different cells, the fact that CAMs represent a very large group of proteins with diverse structural elements predicts that we are only at the beginning of our understanding of the vastly diverse roles that these proteins play in the emergence of neuronal circuits. At the same time, more experiments are needed to elucidate the crosstalk between CAMs and other proteins, such as axon guidance molecules or intracellular signaling components. It will be important to integrate the gained information about individual CAM function to create a more general understanding of cell–cell interaction. Finally, the upstream determinants of CAMs expression in specific neurons and the resulting molecular codes are still largely unknown. These are many challenges that lie ahead to fully understand the function of CAMs not only in visual system development, but also as fundamental strategies of neural circuit formation.

The authors thank Paride Antinucci and Greta Schachermayer for critically reading the manuscript.
